# The Effect of Eccentric Cycling on Cerebral and Muscle Tissue Oxygenation in Patients with Pulmonary Hypertension and Healthy Individuals: A Randomized Controlled Crossover Trial

**DOI:** 10.3390/jcm14165751

**Published:** 2025-08-14

**Authors:** Nico Sturzenegger, Simon R. Schneider, Michael Furian, Anna Titz, Esther I. Schwarz, Mona Lichtblau, Julian Müller, Silvia Ulrich

**Affiliations:** 1 Department of Pulmonology, University Hospital Zurich, 8091 Zurich, Switzerland; nico.sturzenegger@uzh.ch (N.S.); simonrafael.schneider@usz.ch (S.R.S.); michael.furian@usz.ch (M.F.); anna.titz@usz.ch (A.T.); julian.mueller2@usz.ch (J.M.); 2 Faculty of Medicine, University of Zurich, 8032 Zurich, Switzerland

**Keywords:** cardiopulmonary exercise testing, CTEPH, eccentric cycling exercise, PAH, pulmonary hypertension, pulmonary vascular disease, rehabilitation

## Abstract

**Background**: Eccentric cycling exercise (ECC) offers a low-metabolic-demand approach to exercise, potentially making it valuable for patients with pulmonary vascular disease (PVD). The aim of this study was to investigate how quadriceps and frontal cortex oxygenation, assessed by near-infrared spectroscopy (NIRS), differs during ECC compared to concentric cycling exercise (CON) in patients with PVD and in healthy individuals. **Methods**: This randomized controlled crossover trial involved patients with PVD, defined as either pulmonary arterial hypertension (PAH) or chronic thromboembolic pulmonary hypertension (CTEPH), and healthy volunteers. Participants performed both CON and ECC at identical submaximal work rates, following a stepwise incremental protocol. NIRS was used to continuously monitor tissue oxygenation and surrogates for blood volume changes in the quadriceps and frontal cortex. **Results**: A total of 57 participants were included, 33 PVD patients (19 with PAH and 14 with CTEPH; 13 women; mean age: 50 ± 15 years) and 24 healthy volunteers (14 women; 50 ± 14 years). In PVD patients, at end-exercise, cerebral tissue oxygenation (CTO) was significantly higher during ECC compared to CON (6.10%; 95% CI: 1.85 to 10.42; *p* < 0.01), whereas muscle tissue oxygenation (MTO) was similar. In healthy individuals, at end-exercise, CTO was similar during ECC and CON, whereas MTO was significantly higher (2.60%; 95% CI: 0.03 to 5.17; *p* = 0.047). There were no significant differences in CTO and MTO between patients with PVD and healthy individuals. **Discussion**: In this randomized controlled crossover trial, patients with PVD exhibited higher CTO during ECC compared to CON, which may indicate altered cerebral oxygen extraction and hemodynamic responses potentially related to impaired vascular function. In contrast, healthy individuals demonstrated higher MTO during ECC, likely reflecting improved muscular oxygen utilization and efficiency due to the mechanical and metabolic characteristics of eccentric exercise.

## 1. Introduction

Pulmonary vascular diseases (PVDs), including pulmonary arterial hypertension (PAH) and chronic thromboembolic pulmonary hypertension (CTEPH), are characterized by elevated pulmonary vascular resistance, which increases strain on the right ventricle, particularly during exercise, and impairs peak exercise capacity [1]. A hallmark symptom of patients with PVD is exertional dyspnea related to hyperventilation, occurring at rest but especially during exercise [2]. Hyperventilation is driven by increased chemosensitivity, causing increased responsiveness to changes in arterial oxygen (PaO_2_) and arterial carbon dioxide (PaCO_2_) pressure compared to healthy individuals [3,4]. Additionally, hyperventilation is exacerbated by reduced cardiac output and increased physiological dead space [3,5]. These factors contribute to profound exercise intolerance in patients with PVD, characterized by shortness of breath and fatigue, which significantly impair quality of life and lead to inactivity and deconditioning [6]. Although exercise was historically not recommended in patients with PVD, nowadays, evidence supports using structured exercise programs to improve functional capacity and overall well-being [1]. There are ongoing concerns that exercise in patients with PVD would induce deleterious right heart strain and that increased blood flow during exercise could promote pulmonary vascular remodeling and potentially worsen disease progression in patients with PVD. As a result, low-intensity exercise protocols are typically recommended [7,8,9].

During eccentric cycling exercise (ECC), the cyclist works against the backward movement of the pedals on a motor-driven ergometer. During this, working muscles lengthen under load and are more mechanically efficient, resulting in lower energy expenditure for the same workload than conventional concentric cycling (CON) [10,11]. ECC has been shown to reduce oxygen uptake (VO_2_) by up to 80% at identical power output or allow for exercise at higher intensities at an identical metabolic load [12,13,14]. This supports greater exercise tolerance and reduces symptoms like perceived dyspnea. Physiological comparisons between ECC and CON have previously shown that they offer a well-tolerated and effective exercise strategy, especially for patients with cardiopulmonary diseases [1,6,8,13].

Prolonged and regular exercise training leads to chronic adaptations in both ECC and CON contractions. However, the superiority of ECC has recently been demonstrated in a systematic review with meta-analysis. This study found that ECC is more effective than CON because it increases muscle strength, hypertrophy, 6 min walking distance, and, most notably, peak VO_2_, especially in patients with COPD, chronic left heart failure, or coronary heart disease [15].

Therefore, ECC may help maintain muscle and cerebral tissue oxygenation (MTO and CTO), making ECC particularly suitable for patients with PVD, who frequently exhibit exertional oxygen desaturation and limited exercise tolerance [8,16]. Furthermore, CTO might play an important role in perceived dyspnea mechanisms that contribute to exercise limitation in patients with PVD.

This study aimed to investigate how NIRS measurements of quadriceps MTO, frontal cortex CTO, and THb differ during ECC and CON in patients with PVD and healthy individuals.

## 2. Materials and Methods

### 2.1. Study Design, Randomization, Allocation, and Participants

This was a randomized crossover trial at the University Hospital Zurich from February 2022 to August 2023. Randomization was performed using software-based block randomization with variable block lengths determined by random computation. Allocation to different sequences was ensured through allocation concealment in the order of recruitment number. Patients with stable PVD of any sex, aged 18 to 80, diagnosed with either PAH or distal CTEPH according to current guidelines, and with unchanged medication for more than 4 weeks were eligible for inclusion. Patients experiencing severe daytime hypoxemia (PaO_2_ < 7.3 kPa) were excluded. In addition to patients with PVD, the study also included healthy individuals with no cardiopulmonary diseases as controls.

### 2.2. Ethics and Registration

All participants provided written informed consent for participation and for the further scientific use of their data. The study was conducted in compliance with the Declaration of Helsinki, approved by the local ethics committee (KEK 2021-0132), and registered on clinicaltrials.gov (NCT05185895) (NCT05186987). This study followed the Consolidated Standards of Reporting Trials (CONSORT) reporting guideline for crossover trials [17].

### 2.3. Cycling Exercise

Interventions were conducted on two separate days (applied in patients) or with a minimum break of two hours between sessions (applied in healthy participants) to prevent carryover effects. Participants performed two submaximal, standardized, stepwise incremental cycling exercise tests, each with three- to five-minute intervals (totaling 9 to 15 min) at a pedaling rate of 55–65 rpm. One test used a conventional ergometer (Ergoselect 200, Ergoline GmbH, Bitz, Germany), while the other used an eccentric ergometer (Cyclus 2 Recumbent, RBM Elektronik-automation GmbH, Leipzig, Germany), in a randomized order. Exercise intensity began at 50 W and increased by 10 to 30 W per step according to the participant’s fitness level, which was assessed systematically by prior available peak exercise tests or by weekly sport hours. Since the aim of this project was to compare NIRS responses to ECC versus CON at identical work rates, the intra-individual ECC and CON protocols were identical. Peripheral capillary oxygen saturation (SpO_2_) was continuously measured by fingertip pulse oximetry.

### 2.4. Near-Infrared Spectroscopy

Four optodes (two per sensor) were fixed to each patient to measure both MTO and CTO: one sensor on the forehead (prefrontal cortex, to measure cerebral tissue oxygenation) and the other sensor on the vastus lateralis of the quadriceps muscle (to measure muscle tissue oxygenation). These optodes were connected to a NIRS device (Hamamatsu, NIRO-200NX, Shizuoka, Japan). To ensure consistent measurement locations between interventions, a mark was applied. The NIRS technology allows continuous recording of O_2_Hb and HHb in a prespecified tissue volume underneath the optodes. The tissue oxygen saturation [%] was calculated as O_2_Hb/THb (O_2_Hb + HHb). Total hemoglobin (THb) was calculated as the sum of oxygenated (O_2_Hb) and deoxygenated (HHb) hemoglobin signals. To assess dynamic changes during exercise, baseline values were set to zero for each participant, and changes in THb were expressed as relative deviations from this baseline.

### 2.5. Data Presentation and Statistical Analysis

To assess the outcomes of cerebral oxygenated, deoxygenated, and total hemoglobin (cO_2_Hb, cHHb, cTHb) as well as their respective muscle counterparts (mO_2_Hb, mHHb, mTHb) alongside CTO and MTO between CON and ECC at the end of each step, we averaged physiological values from the final 30 s of each step and compared them using repeated-measures statistical analysis. Results are presented as means ± standard errors. A linear mixed model was applied to the data, including interventions (CON vs. ECC), period, and their interaction as fixed effects, while accounting for participant-specific variability through a random intercept. This approach allowed us to account for potential carryover, period effects, and potential confounders, consistent with crossover trial standards. The significance of the intervention–period interaction was tested, and it was omitted from the model if it was not significant. Model assumptions were verified by inspecting residuals and random effects for homogeneity and normality. Missing data were handled within the linear mixed model framework, and an intention-to-treat analysis was performed. Additionally, linear mixed models were used to assess the intervention–group effect for each of the parameters. Statistical significance was defined as a 95% confidence interval excluding the null effect. All computations were conducted using R-Studio software, Version 2024.09.0 + 375.

## 3. Results

In total, 33 PVD patients were included (13 women; mean age 50 ± 15 years; 19 with PAH and 14 with CTEPH), 31 of whom completed both trial periods. One participant dropped out of the study due to coordination problems (ECC), and another was unable to participate in the second phase (CON) for personal reasons (Table 1).

**Figure 1:**
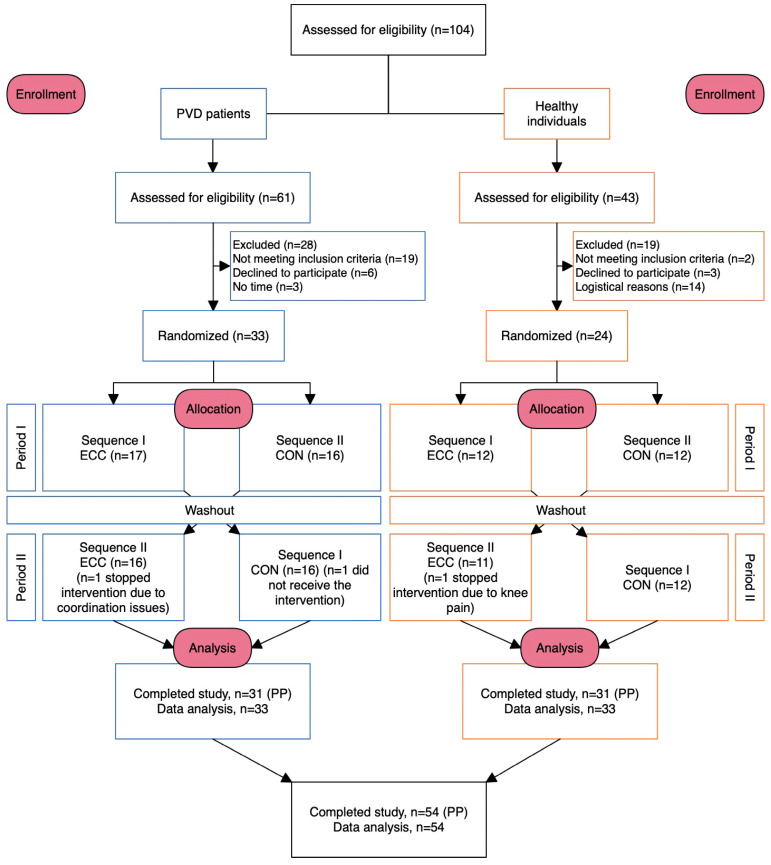
Participant flow chart. CON, concentric cycling exercise; ECC, eccentric cycling exercise; PVD, pulmonary vascular disease; PP, per protocol.

In total, 24 healthy participants were included (14 women; mean age 50 ± 14 years), 23 of whom completed both trial periods. One participant stopped ECC prematurely due to knee pain. The participants’ baseline characteristics are summarized in Table 1, and the study flow is illustrated in Figure 1.

No carryover or period effects were detected in any of the comparisons, neither in patients nor in healthy participants.

### 3.1. Cerebral Tissue Oxygenation and Hemoglobin Concentrations

**Figure 2:**
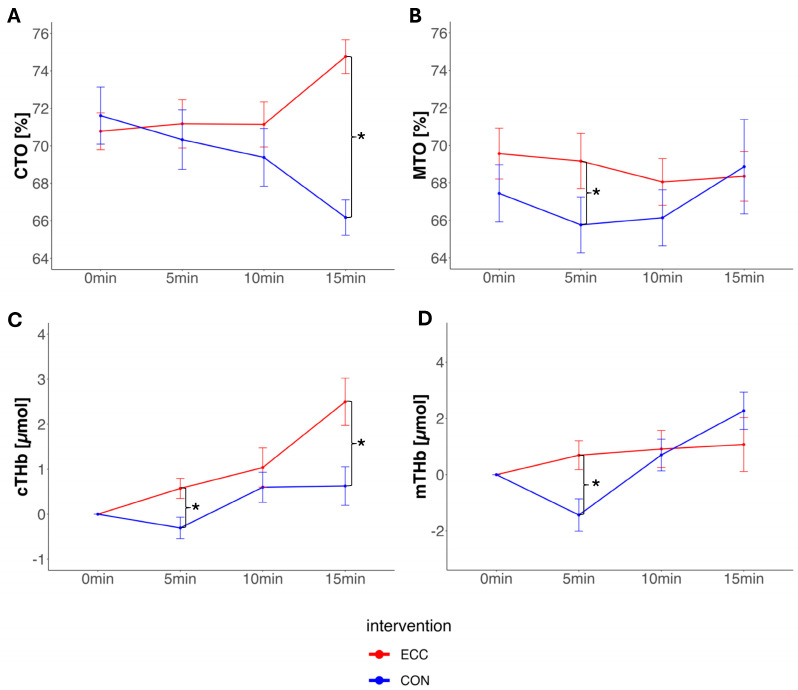
Panels (**A**–**D**): NIRS measurements of the pulmonary vascular disease patients comparing eccentric cycling exercise (red) to concentric cycling exercise (blue). Data are presented as means ± standard errors. * = *p* < 0.05. Panel (**A**): CTO, cerebral tissue oxygenation [%]; panel (**B**): MTO, muscle tissue oxygenation [%]; panel (**C**): cTHb, cerebral total hemoglobin [μM]; panel (**D**): mTHb, muscle total hemoglobin [μM].

In patients, CTO at end-exercise was significantly higher during ECC (74.10 ± 1.90%) compared to CON (68.00 ± 1.90%), with a mean difference of +6.10% (95% CI: 1.85 to 10.42; *p* < 0.01). This was accompanied by significantly higher changes in cTHb with ECC compared to CON (+1.86 μM) (for all 95% CIs and *p*-values, see Table 2 and Table 3), as well as a significant difference in cO_2_Hb compared to CON (+1.94 μM). There was no statistically significant difference in cHHb between interventions at end-exercise (+0.02 μM) (see Figure 2).

**Figure 3:**
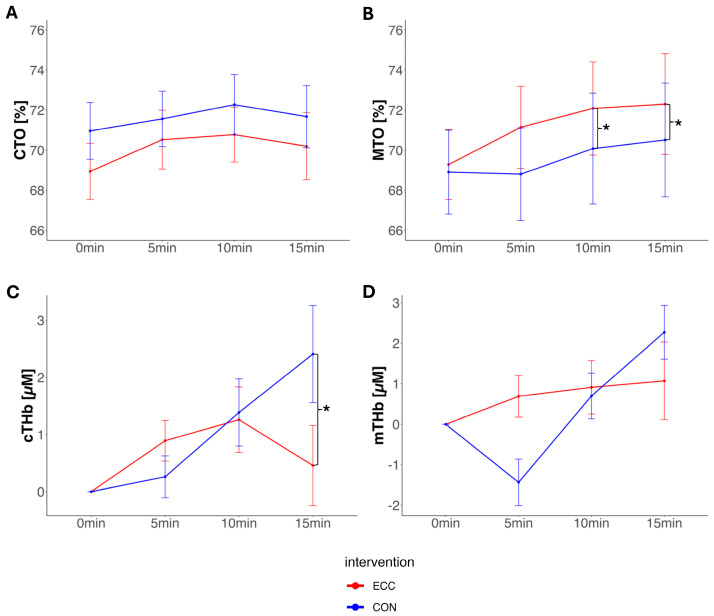
Panels (**A**–**D**): NIRS measurements of the healthy individuals comparing eccentric cycling exercise (red) to concentric cycling exercise (blue). Data are presented as means ± standard errors. * = *p* < 0.050. Panel (**A**): CTO, cerebral tissue oxygenation [%]; panel (**B**): MTO, muscle tissue oxygenation [%]; panel (**C**): cTHb, cerebral total hemoglobin [μM]; panel (**D**): mTHb, muscle total hemoglobin [μM].

In healthy individuals, CTO did not differ significantly between interventions at end-exercise (−1.30; 95% CI: −4.81 to 2.18; *p* = 0.46). Relative to the baseline, cTHb decreased more during ECC than during CON, with a mean difference of −1.94 μM. Differences in cO_2_Hb between interventions at end-exercise were not significant (−0.39 μM). ΔcHHb was significantly lower during ECC compared to CON, with a difference of −1.98μM (see Figure 3).

### 3.2. Muscle Tissue Oxygenation and Hemoglobin Concentrations

In patients, MTO at end-exercise did not significantly differ between ECC and CON (+1.80%; 95% CI: −2.81 to 6.53; *p* = 0.43). Similarly, ΔmTHb showed no significant difference (−1.14 μM), as well as ΔmO_2_Hb (+0.50 μM) and ΔmHHb (−0.89 μM) (see Figure 2).

In healthy individuals, MTO was significantly higher at end-exercise during ECC compared to CON, by 2.60% (95% CI: 0.03 to 5.17; *p* = 0.047). ΔmTHb showed no significant difference (−0.98 μM), nor did ΔmO_2_Hb (+0.09 μM). However, ΔmHHb was significantly lower compared to CON (−1.21 μM) (see Figure 3).

### 3.3. Comparing Patients and Healthy Individuals

The differences between ECC and CON were significantly greater in patients compared to healthy individuals for only two parameters. Specifically, patients exhibited a larger difference in ΔmTHb (+1.46 μM; 95% CI: 0.16 to 2.77; *p* = 0.03) and ΔcHHb (+0.71 μM; 95% CI: 0.22 to 1.20; *p* < 0.01). No statistically significant group differences were found for other parameters.

### 3.4. Peripheral Oxygen Saturation

In patients with PVD as well as in healthy individuals, SpO_2_ at end-exercise did not differ significantly between ECC and CON.

## 4. Discussion

The main findings of this randomized crossover trial in patients with PVD and healthy controls demonstrate, for the first time, that in PVD patients at end-exercise, CTO and relative changes in the THb concentration, a surrogate for blood volume changes (CTO, cO_2_Hb, cTHb), are significantly higher during ECC compared to CON. This provides important insight into potential exercise-limiting factors, such as perceived dyspnea in patients with PVD.

Interestingly, this was not found in healthy individuals, where ΔCTO was similar during ECC and CON, but ΔcTHb was lower during ECC. In the quadriceps muscle, healthy volunteers showed significantly lower ΔmHHb and ΔMTO at end-exercise during ECC compared to CON. Although patients exhibited comparable trends, these differences were not statistically significant. In both PVD patients and healthy individuals, muscle NIRS measurements revealed a stronger increase in ΔmTHb during CON compared to ECC. This suggests that muscle perfusion was more pronounced during CON.

In patients, ECC at end-exercise resulted in significantly higher ΔcTHb and ΔcO_2_Hb compared to CON, while the difference in cHHb was smaller. This suggests that the higher ΔCTO observed during ECC was primarily driven by increased cerebral perfusion, as indicated by the marked rise in ΔcTHb and ΔcO_2_Hb. The relatively small difference in ΔcHHb implies that oxygen extraction efficiency played a smaller role, highlighting perfusion as the dominant factor in maintaining cerebral oxygenation during ECC in PVD patients.

This may be explained by a reduced ventilatory load and lower dyspnea during ECC, which could minimize cerebral vasoconstriction associated with hyperventilation-induced hypocapnia or sympathetic activation [8]. Although absolute cerebral blood flow was not measured, the higher ΔcTHb values suggest improved cerebral hemodynamics under ECC [18].

Patients exhibited a greater initial decrease in cHHb during CON compared to ECC. This might reflect the higher ventilatory demand and enhanced chemosensitivity in PVD patients, leading to a more rapid adjustment in cerebral oxygenation during the onset of CON [3]. However, in the steady state, cHHb levels were higher during CON than during ECC. While the initial response to CON involves a significant decrease in cHHb, the longer-term adjustments in cerebral oxygenation appear less efficient, likely due to the sustained higher energy demands and reduced oxygen delivery efficiency of CON.

The absence of statistically significant differences in muscle NIRS measurements at end-exercise in patients between ECC and CON, unlike in healthy volunteers, may be attributed to the heightened chemosensitivity characteristic of PVD patients. Their increased sensitivity to changes in PaO_2_ and PaCO_2_ can lead to a central dominance in ventilatory regulation, potentially limiting the flexibility of peripheral muscle blood flow in response to eccentric and concentric exercise demands [3]. This phenomenon could attenuate the adaptive differences typically observed in healthy individuals [19].

The distinct dynamics of mHHb between CON and ECC in the patients highlight different physiological adaptations. During CON, the rapid initial decline in mHHb suggests more efficient oxygen extraction driven by higher metabolic demands and the recruitment of fast-twitch (Type II) muscle fibers, which have elevated oxygen consumption [20]. This, combined with transient blood flow restriction from repetitive contractions, amplifies deoxygenation. In contrast, ECC shows a more gradual decline in mHHb, reflecting its lower metabolic demands and reliance on energy-efficient mechanisms like elastic recoil and reduced fiber recruitment, which is in line with known features of ECC [14,21]. In the steady state, persistently higher mHHb during CON underscores the sustained oxygen extraction required for its greater energy demands. ECC, conversely, maintains lower mHHb levels due to superior metabolic efficiency and reduced oxygen consumption.

However, the significant interaction effects suggest distinct adaptations in patients during ECC, such as reduced ΔcHHb and increased ΔmTHb. These may indicate compensatory mechanisms, including altered oxygen extraction and redistribution of blood flow to maintain tissue perfusion under reduced cardiorespiratory reserve [22]. Such adaptations may reflect the unique hemodynamic demands of ECC in PVD, calling for further investigation.

Peripheral SpO_2_ values did not differ significantly between ECC and CON, though a trend towards slightly higher values during ECC was observed. Patients consistently exhibited lower SpO_2_ levels at rest and during exercise compared to healthy individuals [8,9]. Interestingly, despite the absence of significant differences in peripheral SpO_2_, NIRS measurements revealed certain differences in tissue oxygenation between the interventions, suggesting that ECC may influence local tissue oxygenation without substantially altering systemic oxygen saturation. It has been shown previously that eccentric exercise results in higher tissue oxygenation index values compared to concentric exercise, despite no significant changes in SpO_2_ [19,23]. These findings highlight that NIRS is more sensitive to local hemodynamic changes than peripheral pulse oximetry.

### 4.1. Limitations

Limitations of this study include its sample size calculation, which was based on VO_2_ as the main outcome rather than NIRS measurements. The relatively small absolute differences observed in tissue oxygenation likely require a larger sample size to achieve statistical significance. Another limitation is the use of a submaximal exercise protocol, which may not fully capture the physiological responses to maximal exercise capacity. Nevertheless, the approach was necessary due to safety considerations for PVD patients.

### 4.2. Projections

Future studies could conduct a maximal exercise protocol in healthy participants using NIRS to further explore the physiology of ECC. Furthermore, based on our findings, investigations should focus on the role of cerebral oxygenation during exercise and its contribution to exercise limitation, which might be alleviated through specially tailored ECC interventions.

## 5. Conclusions

NIRS measurements revealed differences in muscle and cerebral oxygenation between ECC and CON in both healthy individuals and patients. These findings may contribute to a better understanding of the role of cerebral oxygenation during exercise and its impact on exercise limitation, which could potentially be alleviated through specially tailored ECC interventions, particularly in patients with PVD. This study also highlights the need for further research to elucidate the specific physiological effects of eccentric exercise, such as its influence on dyspnea perception, muscle function, vascular responses, and oxygen utilization, in different patient populations.

## Figures and Tables

**Table 1:** Baseline characteristics. Data are presented as means ± standard deviations or absolute numbers. BMI, body mass index; SpO_2_, peripheral capillary oxygen saturation.

Baseline Characteristics	PVD Patients	Healthy Individuals
Number of participants	33	24
Women; men	13; 20	14; 10
Age [years]	50 ± 15	50 ± 14
BMI [kg/m^2^]	26.7 ± 4.7	23.9 ± 3.3
SpO_2_ [%]	94 ± 3	97 ± 1
Pulmonary arterial hypertension	19	
Idiopathic	11	
Heritable	2	
Associated with the following:		
Congenital heart disease	3	
Connective tissue disease	1	
Portopulmonary hypertension	1	
Veno-occlusive disease	1	
Chronic thromboembolic pulmonary hypertension	14	
Persistent after pulmonary endarterectomy	5	
New York Heart Association functional class, I–III	I: 11, II: 15, III: 7	
6 min walk distance [m]	582 ± 98	
Systolic pulmonary artery pressure at rest [mmHg]	42.5 ± 17.1	
Pulmonary hypertension-targeted medication		
Endothelin receptor antagonist	23	
Phosphodiesterase-5-inhibitor	9	
Prostanoids	5	
Selexipag	3	
Riociguat	12	
Calcium channel blocker	6	
β-blockers	1	

**Table 2:** NIRS results of patients. Data are shown as means ± standard errors, mean differences (plus percentage change (%)), and corresponding 95% confidence intervals. cO_2_Hb, cerebral oxygenated hemoglobin; cHHb, cerebral deoxygenated hemoglobin; cTHb, total cerebral hemoglobin; CTO, cerebral tissue oxygenation; mO_2_Hb, muscle oxygenated hemoglobin; mHHb, muscle deoxygenated hemoglobin; mTHb, total muscle hemoglobin; MTO, muscle tissue oxygenation; SpO_2_, peripheral capillary oxygen saturation.

Baseline PVD Patients 0:00–0:30 min	Eccentric	Concentric	Mean Difference	95% CI	*p*-Value
CTO [%]	70.80 ± 0.98	71.60 ± 1.52			
MTO [%]	69.6 ± 1.36	67.4 ± 1.52			
End-exercise 14:30–15:00 min					
Power output [W]	44 ± 10	44 ± 10			
∆ cO_2_Hb [μM]	2.78 ± 0.50	0.84 ± 0.47	1.94 (230.95%)	0.65 to 3.24	0.004
∆ cHHb [μM]	0.04 ± 0.25	0.02 ± 0.25	0.02 (100%)	−0.65 to 0.68	0.964
∆ cTHb [μM]	2.70 ± 0.50	0.84 ± 0.47	1.86 (221.43%)	0.57 to 3.15	0.005
CTO [%]	74.10 ± 1.90	68.00 ± 1.90	6.10 (8.97%)	1.85 to 10.42	0.005
∆ mO_2_Hb [μM]	3.12 ± 0.67	2.62 ± 0.71	0.50 (19.08%)	−1.41 to 2.41	0.607
∆ mHHb [μM]	−1.33 ± 0.68	−0.44 ± 0.79	−0.89 (−202.27%)	−2.85 to 1.08	0.373
∆ mTHb [μM]	1.08 ± 0.88	2.22 ± 1.03	−1.14 (−51.35%)	−3.77 to 1.48	0.390
MTO [%]	69.50 ± 2.07	67.7 ± 2.21	1.80 (2.66%)	−2.81 to 6.53	0.433
SpO_2_ [%]	92.10 ± 0.56	91.60 ± 0.56	0.50 (0.55%)	−0.65 to 1.51	0.437

**Table 3:** NIRS results of healthy individuals. Data are shown as means ± standard errors, mean differences (plus percentage change (%)), and corresponding 95% confidence intervals (95% CI). cO_2_Hb, cerebral oxygenated hemoglobin; cHHb, cerebral deoxygenated hemoglobin; cTHb, total cerebral hemoglobin; CTO, cerebral tissue oxygenation; mO_2_Hb, muscle oxygenated hemoglobin; mHHb, muscle deoxygenated hemoglobin; mTHb, total muscle hemoglobin; MTO, muscle tissue oxygenation; SpO_2_, peripheral capillary oxygen saturation.

Baseline Healthy 0:00–0:30 min	Eccentric	Concentric	Mean Difference	95% CI	*p*-Value
CTO [%]	68.90 ± 1.40	71.0 ± 1.41			
MTO [%]	69.30 ± 1.74	68.90 ± 2.09			
End-exercise 14:30–15:00 min					
Power output [W]	77 ± 11	77 ± 11			
∆ cO_2_Hb [μM]	2.88 ± 0.52	3.27 ± 0.53	−0.39 (−11.93%)	−1.70 to 0.92	0.555
∆ cHHb [μM]	−2.67 ± 0.31	−0.69 ± 0.31	−1.98 (286.96%)	−2.74 to −1.22	<0.001
∆ cTHb [μM]	0.52 ±0.57	2.46 ± 0.55	−1.94 (−78.86%)	−3.36 to −0.52	0.008
CTO [%]	70.60 ± 1.53	71.90 ± 1.56	−1.30 (−1.81%)	−4.81 to 2.18	0.458
∆ mO_2_Hb [μM]	3.82 ± 0.49	3.73 ± 0.50	0.09 (2.41%)	−1.01 to 1.19	0.87
∆ mHHb [μM]	−2.94 ± 0.49	−1.73 ± 0.48	−1.21 (69.94%)	−2.37 to −0.04	0.043
∆ mTHb [μM]	0.54 ± 0.73	1.52 ± 0.75	−0.98 (−64.47%)	−2.64 to 0.67	0.24
MTO [%]	72.50 ± 2.29	69.90 ± 2.30	2.60 (3.72%)	0.03 to 5.17	0.047
SpO_2_ [%]	95.30 ± 0.36	94.90 ± 0.36	0.40 (0.42%)	−0.24 to 1.04	0.216

## Data Availability

Data are available upon reasonable request to the corresponding author.
